# Supplementary material for: Solid-to-liquid phase transitions of sub-nanometer clusters enhance chemical transformation
Source: Nat Commun. 2019 Nov 27;10:5400. doi: 10.1038/s41467-019-13509-3 (PMC6881434; doi:10.1038/s41467-019-13509-3)
Supplement: Supplementary file 2 — Supplementary Information [file 41467_2019_13509_MOESM2_ESM.pdf]

Supporting Information

Solid-to-liquid phase transitions of sub-nanometer clusters enhance chemical transformation

Juan-Juan Sun, Jun Cheng*

Collaborative Innovation Center of Chemistry for Energy Materials (*i*ChEM), State Key Laboratory of Physical Chemistry of Solid Surfaces, College of Chemistry and Chemical Engineering, Xiamen University, Xiamen 361005, China.

Table of Contents

Supplementary Methods 2

Supplementary Figures. 1-11........ 4

Supplementary Tables 1-2........ 15

Supplementary References........ 17

Supplementary Methods

Free Energy Calculation. These free energy calculation methods are well established, and often used in solution reactions[1–4] and biological processes[5]. The detailed formulation of the method can be found in literature[6,7]. Briefly, by taking the derivative of the free energy $A(\xi)$ with respect to the reaction coordinate ξ one can obtain[7] $\frac{dA}{d\xi} = \langle \frac{\delta H_{\xi}^{\mathrm{F}}}{\delta \xi} \rangle$, where H_{ξ}^{F} is the Fixman Hamiltonian of the generalized coordinates. The derivative of the Hamiltonian with respect to ξ can be seen as an external force which is applied on the system to keep the reaction coordinate constant. In our calculations, the change of the Hamiltonian with respect to ξ is expressed as the Lagrange multiplier λ for constraining the distance[6]. Thus, to calculate the free energy of O_2 dissociation, we first performed constrained MD simulations to calculate the mean forces (i.e. Lagrange multipliers) for a set of O-O distances along the reaction coordinate from the reactant (e.g. O_2 molecule on Au_{13}) to product state (e.g. two separate O atoms on Au_{13}). In the constrained MD runs, we gradually increased the O-O distance with small increments, using the last structure of the previous MD run as the initial configuration for the next run. The free energies were then obtained by integrating the average forces with respect to the distance.

To ensure the accuracy, we carried out extensive AIMD runs. It is worth mentioning: (i) it takes about 10-20 ps, i.e. 20000-40000 AIMD steps, to converge one mean force; (ii) we calculated overall 15 distances along the reaction coordinate for O_2 dissociation on Au_{13}, amounting to about half million AIMD steps to obtain one free energy profile; (iii) to further study the temperature effect, we investigated 7 temperatures well covering the temperature range of the solid, solid-liquid coexistence and liquid phases. Thus, it overall takes about 4-5 million AIMD steps to obtain sufficient data to assess the temperature dependence of the reaction free energies.

Estimate of Statistical Errors in Free Energy Calculation. To quantify the statistical errors on the calculated PMFs as well as the free energies, we have divided the MD trajectories into five evenly-spaced blocks, and calculated the average PMFs and free energies in separate, and then used the standard deviations of these five blocks of data as measure of statistical uncertainties. Consistent with good convergence of time accumulating averages (Supplementary Fig. 3a and b), the estimated statistical uncertainties are indeed negligible, on the order of 0.03 eV $Å^{-1}$ for forces and 0.01 eV for energies (Supplementary Fig. 3e and f). The error bar in the entropies, obtained by differentiating free energies with respect to the temperature, is however not straightforward to estimate. Note that it is not a statistical error, but a fitting error. The fitting at low and high temperature ranges are certainly more accurate, because of slowly varying of free energies, than that at the transition temperature range where free energies change more dramatically (Fig. 1c). The fitting in the transition range will benefit from calculating more free energy points. If simply taking the two points at 273 K and 373 K and assuming a linear fit, the free energy change (Supplementary Table 1 for detailed values) is about 1.2 eV, giving an average entropy change of ~1200 J mol^{-1} K^{-1} . This is of similar magnitude to the peak in the curve fitting. Most importantly,

the sharp entropy change with such a magnitude clearly indicates the occurrence of phase transitions, irrespective of the fitting procedure of temperature dependent free energies.

	**a**	**b**	**c** front view top view
	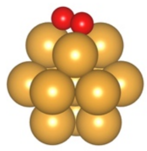	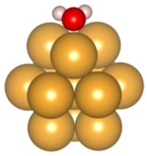	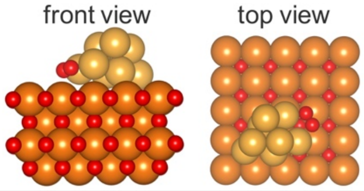
E_{ads}	-0.54 eV	-0.42 eV	-0.34 eV
O-O bond lengths	1.36 Å	—	1.35 Å

Supplementary Figure 1. Model structures. **a** O_2 on Au_{13}. **b** H_2O on Au_{13}. **c** O_2 on Au_8/MgO(001). The inset values show the corresponding adsorption energies and O-O bond length. The balls in yellow, red, pink and orange represent Au, O, H and Mg atoms, respectively.

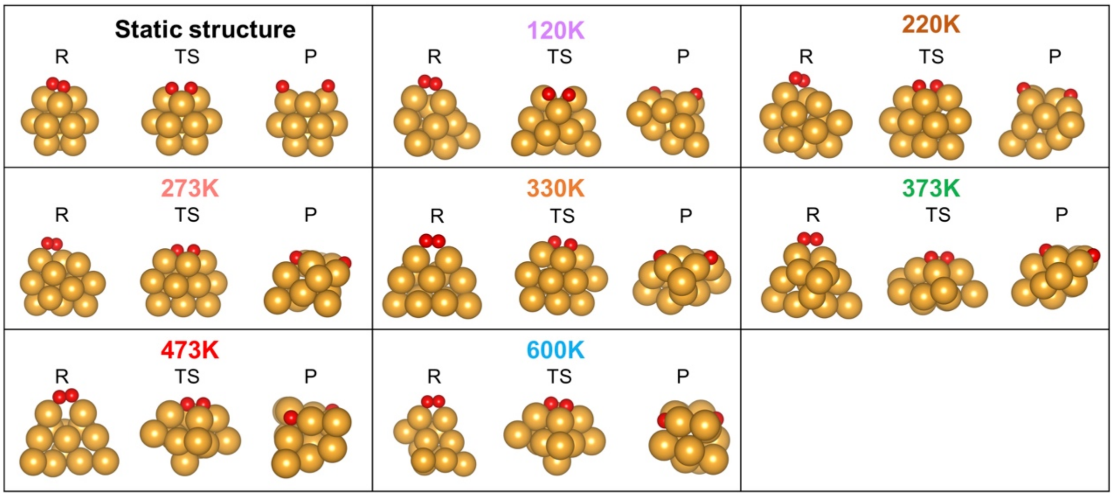

Supplementary Figure 2. Structures of O_2 dissociation on Au_{13} calculated by static geometry optimization (0 K) and using AIMD under finite temperatures. R, TS and P denote the reactant, transition state and product state, respectively. The balls in yellow and red represent Au and O atoms, respectively.

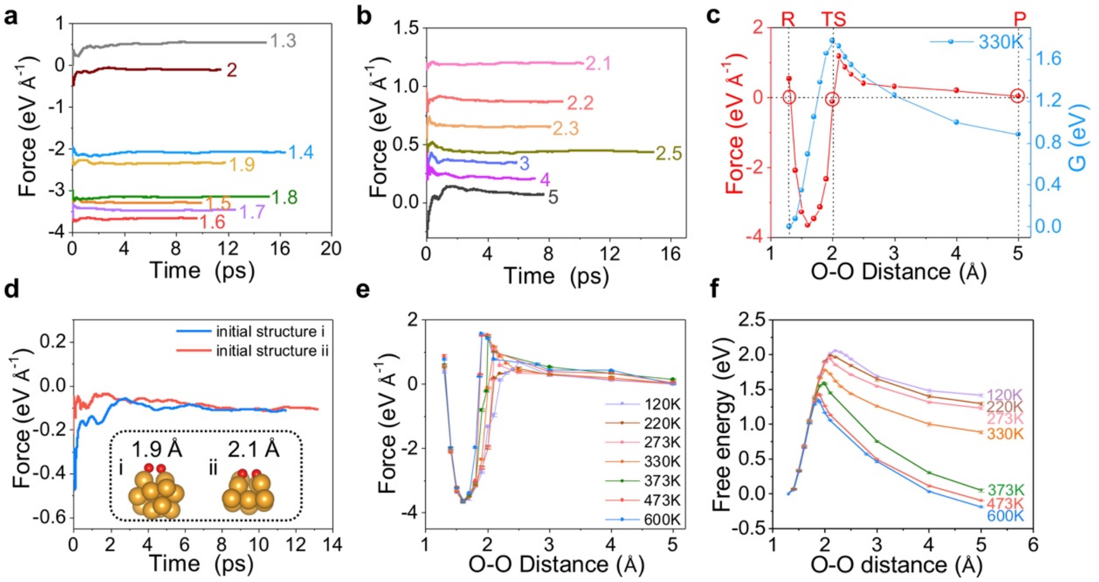

Supplementary Figure 3. Potential of mean force calculation of O_2 dissociation on Au_{13}. **a** Time accumulative averages of forces at the O-O distance varying from 1.3 Å to 2.0 Å and **(b)** from 2.1 Å to 5.0 Å at 330 K. The inset values show the corresponding O-O distances. **c** The red curve shows the averaged force-distance curve at 330 K. The three points corresponding to zero force from left to right correspond to the reactant, TS and product, respectively. The integration of the averaged force-distance curve leads to the free energy profile (blue curve). **d** Time accumulative averages of forces at the O-O distance of 2.0 Å with different initial structures. Insert shows snapshots of the structures of Au_{13}-O-O with different O-O distances. i, O-O distance is 1.9 Å. ii, O-O distance is 2.1 Å. The balls in yellow and red represent Au and O atoms, respectively. **e** Calculated averaged force-distance curves with error bars under various temperatures. **f** Free energy profiles with error bars for O_2 dissociation on Au_{13} under different temperatures.

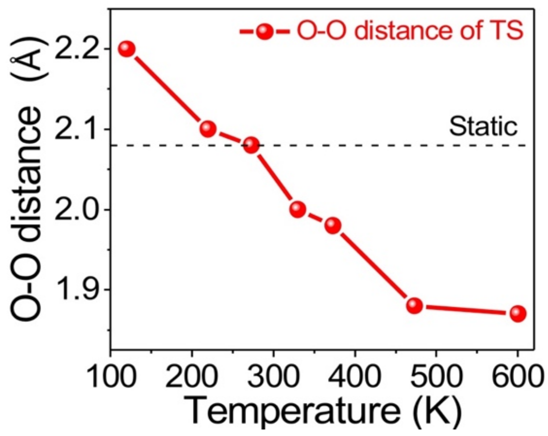

Supplementary Figure 4. O-O distances at the transition state (TS) of the O_2 dissociation on Au_{13}. The O-O distances at TS calculated using AIMD at various temperatures are compared with the one obtained using static geometry optimization.

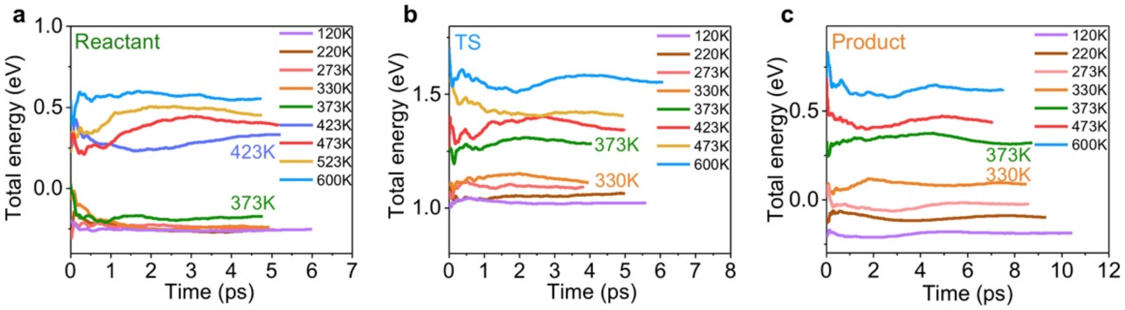

Supplementary Figure 5. Convergence of time averages of total energies of O_2 dissociation on Au_{13} under various temperatures. **a** Reactant. **b** TS. **c** Product. The inset indicates the corresponding temperatures.

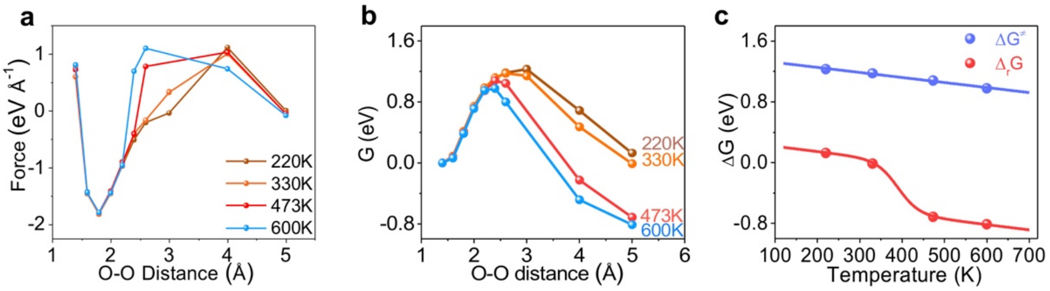

Supplementary Figure 6. Free energy calculation of O_2 dissociation on Au_8/MgO by AIMD. **a** The force-distance curves under different temperatures. **b** The free energy profiles under different temperatures. The inset shows the corresponding reaction temperatures. **c** Temperature dependence of reaction free energy ($\Delta_r G$) and free energy barrier ($\Delta G^{\ddagger}$). The dots show the calculated values and the lines are fitting curves.

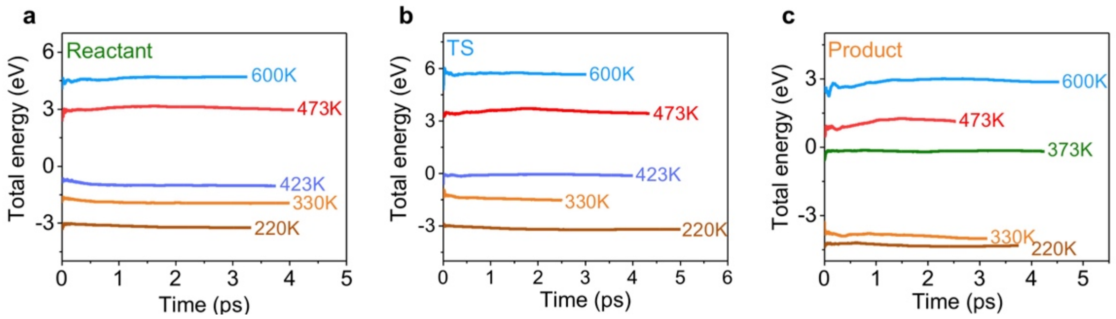

Supplementary Figure 7. Convergence of time averages of total energies of O_2 dissociation on Au_8/MgO under different temperatures. **a** Reactant. **b** TS. **c** Product. The inset shows the corresponding temperatures.

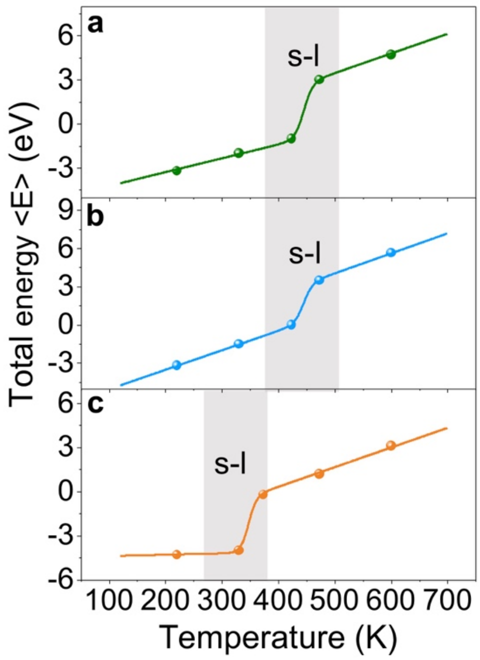

Supplementary Figure 8. Canonical caloric curves ⟨E⟩(T) of O_2 dissociation on Au_8/MgO. **a** Reactant. **b** TS. **c** Product. The dots show the calculated values and the lines are fitting curves. The s-l indicates solid-liquid coexistence state.

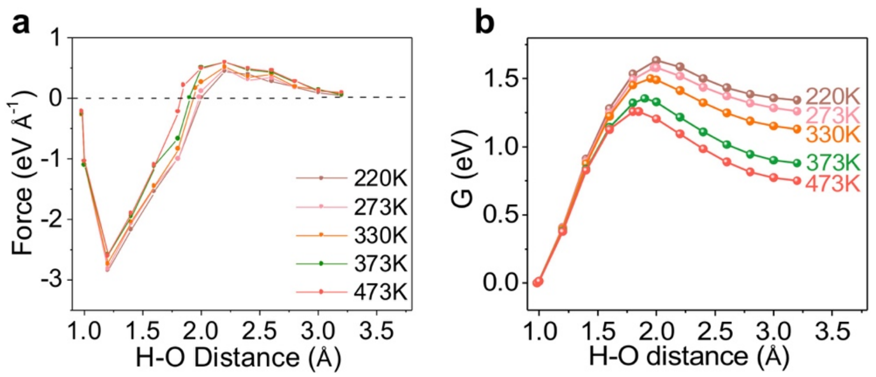

Supplementary Figure 9. Free energy calculation of H_2O dissociation on Au_{13}. **a** The force-distance curves under different temperatures. **b** The free energy profiles under different temperatures. The inset indicates the corresponding temperatures.

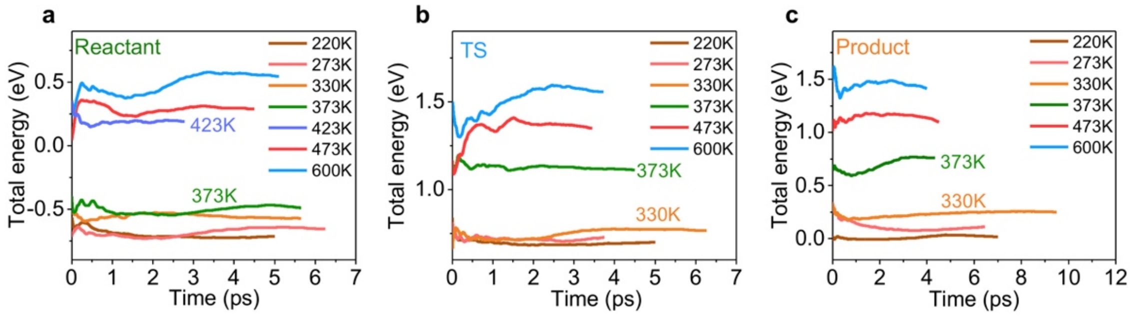

Supplementary Figure 10. Convergence of time averages of total energies of H_2O dissociation on Au_{13} under different temperatures. **a** Reactant. **b** TS. **c** Product. The inset shows the corresponding temperatures.

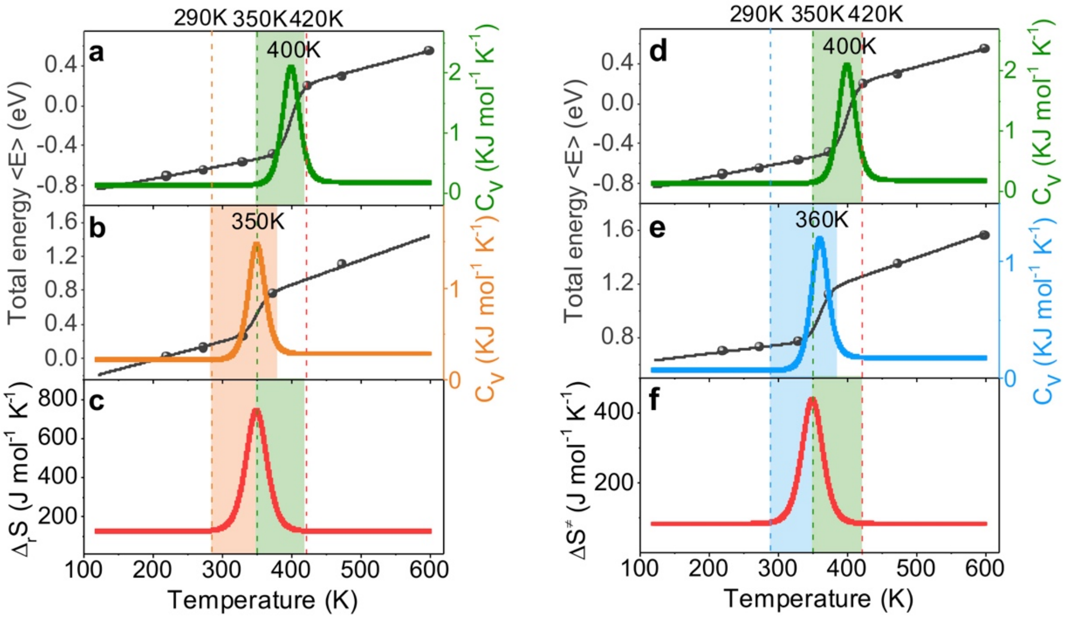

Supplementary Figure 11. Correlation of phase transition behaviors and entropy changes of H_2O dissociation on Au_{13}. **a** The ⟨E⟩(T) curve and C_v(T) curve (green) of the reactant. **b** The ⟨E⟩(T) curve and C_v(T) curve (orange) of the product. **c** Temperature dependence of the reaction entropy change ($\Delta_r S$). **d** The ⟨E⟩(T) curve and C_v(T) curve (green) of the reactant. **e** The ⟨E⟩(T) curve and C_v(T) curve (blue) of TS. **f** Temperature dependence of the activation entropy ($\Delta S^{\ddagger}$). The corresponding temperature at the maximum C_v is defined as the melting point (T_m) indicated as insert values. The C_v(T) can be divided into three regions, corresponding to the different phases. The dots show the calculated values and the lines are fitting curves.

Supplementary Table 1. Temperature dependence of reaction free energies ($\Delta_r G$) and free energy barriers ($\Delta G^{\ddagger}$) of O_2 dissociation on Au_{13} calculated by AIMD.

Temperature (K)	120	220	273	330	373	473	600
Free energy barrier (eV)	2.05	1.99	1.95	1.78	1.59	1.44	1.35
Reaction free energy (eV)	1.41	1.30	1.23	0.89	0.05	-0.09	-0.19

Supplementary Table 2. Temperature dependence of reaction free energies ($\Delta_r G$) and free energy barriers ($\Delta G^{\ddagger}$) of O_2 dissociation on Au_{13} calculated using static geometry optimization (0K). The entropic (i.e. vibrational) contributions were corrected using the harmonic approximation.

Temperature (K)	120	220	273	330	373	473	600
Free energy barrier (eV)	1.58	1.6	1.61	1.62	1.63	1.64	1.66
Reaction free energy (eV)	0.40	0.40	0.40	0.40	0.40	0.40	0.40

Supplementary References

1. Ciccotti, G., Ferrario, M., Hynes, J. T. & Kapral, R. Constrained molecular dynamics and the mean potential for an ion pair in a polar solvent. *Chem. Phys.* **129,** 241–251 (1989).
2. Ciccotti, G., Ferrario, M., Hynes, J. T. & Kapral, R. Dynamics of ion pair interconversion in a polar solvent. *J. Chem. Phys.* **93,** 7137 (1990).
3. Keirstead, W. P. & Wilson, K. R. Molecular dynamics of a model SN1 reaction in water. *J. Chem. Phys.* **95,** 5256 (1991).
4. Curioni, A. et al. Density functional theory-based molecular dynamics simulation of acid-catalyzed chemical reactions in liquid trioxane. *J. Am. Chem. Soc.* **119,** 7218–7229 (1997).
5. Boczko, E. M., Brooks, C. L. First-principles calculation of the folding free energy of a three-helix bundle protein. *Science* **269,** 393–396 (1995).
6. Sprik, M. & Ciccotti, G. Free energy from constrained molecular dynamics. *J. Chem. Phys.* **109,** 7737–7744 (1998).
7. Chipot, C. & Pohorille, A. Editors free energy calculations. *Springer Ser. Chem. Phys.* (2007).
